# National Dental Association

**Published:** 1898-02

**Authors:** 


### NATIONAL DENTAL ASSOCIATION.

(Continued from page 48.)

*August 6, 1897.*

THE meeting was called to order by President Fillebrown, at one o'clock, in the ball-room of the Hotel Chamberlain.

Dr. Walker read the minutes of the previous session, up to the time of consolidation and election of officers, after which Dr. Cushing continued the reading of the minutes. The same were duly approved.

The Secretary then read the organization of committees as follows:

*Executive Committee.*—J. N. Crouse, chairman; M. F. Finlay.

*Committee on Arrangements.*—Drs. Crouse, Noel, and Smith.

*Auditing Committee.*—Drs. Jackson, Patterson, and Finlay.

*Committee on Voluntary Essays.*—Drs. Peirce, Dickinson, and Eubank.

*Committee on Publication.*—Drs. Harlan, Turner, and Cushing.

*Committee on National Museum and Library.*—Drs. Donnelly, Morgan, and Fillebrown.

*Committee on Terminology.*—Drs. Guilford, Molyneaux, and Foster.

*Committee on Journal.*—Drs. Marshall, Taft, and Noel.

*Committee on History.*—Drs. McManus, R. F. Hunt, and Gordon White.

OFFICERS OF THE VARIOUS SECTIONS.

SECTION I.—Grant Molyneaux, chairman; R. R. Freeman, secretary.

SECTION II.—Dr. Catching, chairman; Dr. Finlay, secretary.

SECTION III.—Dr. Crawford, chairman; Dr. Holland, secretary.

SECTION IV.—Dr. J. I. Hart, chairman; Dr. Hinman, secretary.

SECTION V.—Dr. J. S. Cassidy, chairman; Dr. L. P. Bethel, secretary.

SECTION VI.—J. D. Patterson, chairman; L. E. Custer, secretary.

SECTION VII.—W. C. Barrett, chairman; George B. Clement, secretary.

*Hygiene and Prophylactic Dentistry.*—Dr. Noel, chairman; Dr. Thompson, secretary.

*Orthodontia.*—Dr. Jackson, chairman; Dr. Dotterer, secretary.

*Clinics.*—Dr. McKellops, chairman; Dr. E. P. Beadles, secretary.

Dr. Crouse presented the following, which was moved as a standing resolution:

*Resolved*, That any member of the dental profession who has been in reputable practice for a period of fifty years may be elected to permanent membership in this Association without the payment of dues, and any member of this Association who has been in practice for a like period shall have his dues remitted thereafter, by presenting the fact to the Association.

Carried.

Dr. Finlay offered the following resolution:

*Resolved*, That the Committee on National Dental Library and Museum be authorized to seek, in the interest of public health, the employment at government expense of at least one dentist of eminent fitness in the Army Medical Library and Museum at Washington, whose time when so employed shall be devoted to the advancement of dental science.

Motion carried.

Dr. Taft offered the following resolution:

*Resolved*, That a committee of three shall be appointed, whose duty it shall be to confer with State and local societies, and aid and foster new societies when and where desirable.

Carried.

*Dr. Cushing.*—It was suggested, as to the publication of the transactions, that it would be a good thing for both societies to print as a supplement to their transactions the transactions of this National Dental Association, provided this Association will consent to that.

*Dr. Crawford.*—I move that the National Dental Association request the officers of the American Dental Association and the officers of the Southern Dental Association to incorporate in their published reports all of the proceedings of the National Dental Association since its organization yesterday.

Carried.

*Dr. Taft*—In reference to the resolution I just offered, I would say that such a committee can be eminently serviceable to the profession, to local societies especially, being known as the connecting link between this society and all other societies in the country. We hope that this committee will make a report at the next annual meeting.

*Dr. Crouse.*—I move that a committee be appointed from this Association, consisting of one member from each State and Territory, to work in the interests of the Dental Protective Association, and to see that the delegates are members of the Dental Protective Association.

Motion carried.

(The appointment of such committee will appear in the printed transactions.)

NEW BUSINESS.

*Dr. Richards.*—In consideration of the fact that the American Dental Association and the Southern Dental Association have passed into history, I move that the gavels of the two Associations be properly engraved and stored away in the archives at Washington, together with the other effects that we have voted to go there.

Motion carried.

*Dr. Crawford.*—I want to ask unanimous consent to do one more thing before we adjourn, and the reason I ask it is because I know it is right, and will result in the most good to the dental profession in the United States. When there is any one definite thing to be done of public interest to everybody there is usually a very easy way to find out how it ought to be done. The American Dental Association has acted gracefully, and the Southern Dental Association has acted gracefully and nobly. The members of these two historic organizations have come together in the proper fraternal spirit, and we have to-day completed the greatest organization of dental surgeons that is now in the world. If we do our duty, future history will have it that it is the greatest organization that this century has any cognizance of; but if we act timidly when a responsibility is placed upon us, the future will not be so glorious. The old American Dental Association has folded its banner, and, like the Arab, is preparing to "silently steal away," and the Southern Dental Association will have a meeting in Florida one year from now and maintain its identity and its name. In view of all this, I rise for the good of the profession to ask unanimous consent that in deference to the previous history of American dentistry, and the devotion of every man to the Union and the constitution, the name of this Association be "The American Association of Dental Surgeons."

*The President.*—The chair feels obliged to rule that he cannot entertain that motion for unanimous consent. Had that appeared in the convention before they were acting under its constitution, it would have been perfectly permissible, and the name could have been changed. The Association organized, took its constitution for a guide, elected its officers, adjourned, and now meets in a second session. It seems to me that it would be incompetent to entertain that motion and change the name.

*Dr. Moore.*—Is it not competent for this body to reconsider any action that it has taken at the first meeting?

*The President.*—I should think not, because so many are absent from this meeting who were present at the last.

*Dr. Stellwagen.*—I feel that I am among intimate friends and men who have known me for years. Some differ with me, but I sincerely believe, if they will think the subject over thoroughly, that there is a way out of all trouble in regard to the name. We have had three great epochs in this nineteenth century in dentistry. We have embraced a study of the mouth entirely, and not confined ourselves to teeth. We have now united two great societies, and as a result of the union of these two societies the time seems to be propitious for the adoption of the name which is truly explanative of what we are,—stomatologists. We no longer confine ourselves simply to the teeth, and send our patients who require attention with regard to the soft tissues of the mouth or jaws to other men. We have taken up the study of the entire mouth, and the work of these two societies has been stomatological, and not merely dental. It is a narrow term which does not encompass the entire work. I move as an amendment, that we may act upon it coolly and deliberately at our next annual meeting, that the word "stomatological"

be inserted in the place of the word "dental." That would make it the "American Stomatological Association." We are yet in swaddling clothes, but it is the swaddling clothes of a broader and more complete education and profession, with no disrespect to dentistry. It is time we have grown out of the old rut.

*Dr. Peirce.*—I hope the suggestion just made will not be considered, and we shall not make ourselves the subject of ridicule by all scientific men. "Stoma" means an opening, not the oral cavity at all. It has no more relation to the mouth than to any other cavity or opening in the body.

*Dr. Hunt.*—We cannot make any change at this session of the National Dental Association without the unanimous consent of all members. I give notice that I will oppose at this session any change in the name. I do so for the best interests of the Association, and I want to prevent any hasty action. We have this National Dental Association; we came here to form it, and now do not let us change it.

*Dr. Crawford.*—I move that the secretary be requested to read the resolution that was presented in conformity with the constitution and by-laws, and that we now proceed to properly name this organization.

*The President.*—The chair has knowledge that there will be objections raised and persisted in.

*Dr. Guilford.*—I would like to offer an amendment. Dr. Crawford proposed the name of "The American Association of Dental Surgeons;" Dr. Stellwagen proposed the name of "American Stomatological Association." I would suggest the name of the "American Academy of Dental Science." An eminent professor, in conferring a degree, said, instead of "Doctor of Dental Surgery," "Doctor of Dental Science," and when his attention was called to it, he contended that he had not made any mistake, and that he had stated it correctly.

Adjournment.

